# Drug-resistant tuberculosis in Colombia, 2013-2018: Case-control study

**DOI:** 10.7705/biomedica.6842

**Published:** 2023-12-01

**Authors:** Gloria Mercedes Puerto, Claudia Marcela Castro, Vivian Vanesa Rubio, Santiago Fadul, Fernando Montes

**Affiliations:** 1 Grupo Micobacterias, RED TB COLOMBIA, Dirección de Investigación en Salud Pública, Instituto Nacional de Salud, Bogotá, D. C., Colombia Dirección de Investigación en Salud Pública Instituto Nacional de Salud Bogotá, D. C. Colombia; 2 Equipo Banco de Proyectos, RED TB COLOMBIA, Dirección de Investigación en Salud Pública, Instituto Nacional de Salud , Bogotá, D. C., Colombia Dirección de Investigación en Salud Pública Instituto Nacional de Salud Bogotá, D. C. Colombia; 3 Grupo Micobacterias, RED TB COLOMBIA, Dirección de Vigilancia y Análisis del Riesgo en Salud Pública, Instituto Nacional de Salud, Bogotá, D. C., Colombia Dirección de Vigilancia y Análisis del Riesgo en Salud Pública Instituto Nacional de Salud Bogotá, D. C. Colombia; 4 Programa de Tuberculosis, RED TB COLOMBIA, Secretaría de Salud de Medellín, Colombia Secretaría de Salud de Medellín Colombia

**Keywords:** tuberculosis, drug resistance, multiple, risk factors, retrospective studies, comorbidities, case-control studies, black people, tuberculosis, resistencia a múltiples medicamentos, factores de riesgo, estudios retrospectivos, comorbilidad, estudios de casos y controles, población negra

## Abstract

**Introduction.:**

Multidrug-resistant/rifampicin-resistant tuberculosis (MDR/RR-TB) is difficult to control, has high morbidity and mortality, and demands priority public health intervention. In Colombia, MDR/RR-TB has been becoming more widespread annually. Before the COVID-19 pandemic, over an 8-year period, the number of cases of multidrug-resistant tuberculosis in Colombia was close to a thousand cases. Timely identification of the different risk factors for MDR/RR-TB will contribute fundamentally to the systematic management.

**Objective.:**

To determine which risk factors were associated with the presentation of MDR in Colombia between 2013 and 2018.

**Materials and methods.:**

A retrospective case-control study was carried out, for which the data from the routine surveillance of MDR/events in the country were used.

**Results.:**

The cases of multidrug-resistant tuberculosis were mainly in young people, Afrodescendants, and males. Of the clinical conditions, comorbidities such as malnutrition, diabetes, and HIV, presence of at least one factor, such as drug dependence, taking immunosuppressive medications, belonging to the black race, afro, and living in an area of high disease burden were risk factors.

**Conclusion.:**

In addition to the diagnosis and timely provision of MDR-TB treatment, it is necessary that public health programs at the local level pay special attention to patients with the identified risk factors.

## Resumen

**Introducción.:**

La tuberculosis multirresistente-resistente a la rifampicina (TB-MDR/RR) es difícil de controlar, tiene una alta morbilidad y mortalidad y exige una intervención prioritaria en salud pública. En Colombia, la TB-MDR/RR se ha ido extendiendo cada año. Antes de la pandemia de COVID-19, en un periodo de 8 años, el número de casos de TB-MDR/RR en Colombia se acercaba a los mil. La identificación oportuna de los diferentes factores de riesgo de TB-MDR/RR contribuirá de manera fundamental al manejo sistemático de la enfermedad.

**Objetivo.:**

Determinar los factores de riesgo que se asociaron a la presentación de la TB- MDR/RR en Colombia entre 2013 y 2018.

**Materiales y métodos.:**

Se realizó un estudio retrospectivo de casos y controles, para el cual se utilizaron los datos de la vigilancia rutinaria de eventos de TB MDR/RR en el país.

**Resultados.:**

Los casos de TB MDR se presentaron principalmente en jóvenes, afrodescendientes y varones. De las condiciones clínicas, fueron factores de riesgo las comorbilidades como la desnutrición, la diabetes y el VIH, y la presencia de, al menos, un factor como la farmacodependencia, el consumo de medicamentos inmunosupresores, el ser de raza negra o afro y el vivir en una zona del país de alta carga de tuberculosis.

**Conclusiones.:**

Además del diagnóstico y la provisión oportuna del tratamiento de la TB MDR, es necesario que los programas de salud pública a nivel local presten especial atención a los pacientes con los factores de riesgo identificados.

Tuberculosis is the 13th-leading cause of death worldwide, with a greater impact in low-income countries, with a death rate just behind that of COVID-19 ^1^. In 2020, 1.5 million people died and 10 million developed tuberculosis ^2^. Multidrug-resistant/rifampicin-resistant TB (MDR/RR-TB) is considered a public health crisis. MDR TB is caused by an organism that is resistant to at least isoniazid and rifampin, the two most potent tuberculosis drugs used to treat all persons with disease. In 2020, there were 500,000 cases globally resistant to rifampicin, of which 132,222 were MDR/RR-TB. An alarming situation is that between 2018 and 2019, only 333,304 people with MDR/RR-TB were treated, which is 22% of the goal proposed by the World Health Organization (WHO) ^3^. This has been aggravated by the COVID-19 pandemic; it is estimated that there has been a decrease of 15% in people who enter treatment ^(^^1^.

In Colombia, MDR/RR-TB has been becoming more widespread: From 2012 to 2019, 976 cases were reported to the Epidemiological Surveillance System. The number of MDR/RR-TB cases estimated in Colombia by the WHO for 2019 was 610, with a national incidence of 1.2 per 100,000 inhabitants ^3^, however, there were only 239 reported cases ^4^. The onset of the COVID-19 pandemic has caused important health gaps, and the WHO predicts that the number of people diagnosed and treated for tuberculosis may decrease from 25 to 50%, setting the advancement of the control of the disease back by as many as 8 years ^3^.

Some risk factors for MDR/RR-TB have been described: a history of previous treatment, exposure to a known patient with MDR/RR-TB, living in high-prevalence places, and living in conglomerate centers such as prisons and hospitals ^4^^-^^6^. The knowledge of all the risk factors is a pillar for the systematic management of the disease, according to the characteristics of each region.

The main objective of the present study was determining which risk factors were associated with the presentation of cases of MDR reported to the surveillance system in Colombia between 2013 and 2018.

## Materials and methods

This retrospective analytical observational case-control study was conducted based on data from routine surveillance of the MDR-TB event, collected by the Colombian *Instituto Nacional de Salud* from 2013 to 2018.

### Study population

The data was extracted from a secondary database of patients who entered the National Tuberculosis Control Program. A database in Microsoft Excel 2010® was filled out.

*Case:* patient with confirmed pulmonary tuberculosis diagnosis and laboratory-confirmed resistance to rifampicin and isoniazid.

*Control:* patient with a confirmed diagnosis of tuberculosis that was sensitive to treatment with rifampicin and isoniazid, verified by laboratory. This control group was randomly selected and was paired both by the year of notification and by place of residence in which the cases were identified. At least one control was included for each case, considering only those patients with complete data for analysis.

*Control of biases and confounding variables*: This was done at two times: 1) Before conducting the study: a) by pairing the cases with the controls as described in the previous paragraph; b) by including only patients with a laboratory-confirmed diagnosis of MDR to control for selection bias; and c) by randomization of the respective controls. 2) Once the data were obtained, the possible confounding variables were controlled by a multivariate logistic regression analysis.

### Statistical analysis

Descriptive statistics were performed. For variables measured on a nominal scale, the analysis was performed using absolute frequencies and proportions. The variables measured on the numerical scale were categorized to analyze them as described for the variables measured on the nominal scale.

All demographic and clinical variables were obtained from the reports to the surveillance system of the Colombian *Instituto Nacional de Salud*. The definitions were taken from the notification sheets ^7^^,^^8^. A descriptive analysis was performed on the demographic and clinical characteristics of all selected cases and controls: sex, age, ethnicity, department of residence, history of anti-tuberculosis treatment, body mass index (BMI), and exposure to any of the following factors: 1) being drug dependent; 2) taking immunosuppressive medications; 3) living in areas with a high burden of drug-resistant tuberculosis; and 4) presence of some comorbidity (malnutrition, diabetes, HIV, silicosis, kidney disease, liver disease, cancer, or arthritis). Bivariate analysis was performed to search for associations between the different variables measured on a nominal scale and MDR -TB by means of Pearson’s x^2^ test.

Odds ratios (OR) with their 95% confidence intervals (CI) were calculated to measure the association between the exposure variables and the presence of MDR-TB. A value of p≤0.05 was considered statistically significant ^9^^,^^10^. With the variables identified in the bivariate analysis as potential confounders, in addition to those that, due to biological plausibility, should enter the analysis, a binary multivariate logistic regression was performed by the enter (backward) method to evaluate the possible risk factors for MDR-TB. The fit of the model was evaluated by the likelihood ratio. The percentage of MDR/RR- TB explained by each risk factor was estimated by the Nagelkerke test ^10^.

The data analysis was performed using the statistical package Stata^™^, version 16 (Stata Corp. 2019, Stata Statistical Software: Release 16. College Station, TX: Stata Corp LLC.) A significance of 95% was considered for all tests, that is, a type I error of 5%; therefore, a value of p≤0.05 was set as significant.

### Ethical considerations

This research was approved by the Research Ethics and Methodology Committee of the Colombian *Instituto Nacional de Salud* 15-2015.

## Results

In Colombia, from 2013 to 2018, there were 80,601 cases of tuberculosis, of which 597 (0.74%) were MDR-TB. The departments of Antioquia and Valle del Cauca were home to more than 50% of MDR-TB cases, followed by Risaralda and Bogotá. The total number of MDR-TB cases by year of occurrence is presented in table 1.

**Table 1:** MDR-TB cases at the national level per year, Colombia, 2013 to 2018

Year	n	%
2013	72	12.06
2014	92	15.41
2015	84	14.07
2016	114	19.10
2017	131	21.94
2018	104	17.42
Total	597	100

A total of 2,045 patients were analyzed in this study (597 MDR-TB cases and 1,448 controls). During the period analyzed, the same year-to-year trend was presented, no significant change was observed. The male sex was the most affected, with 63.9%. The cases were more concentrated in the 25- to 54-year-old group. A total of 59.8% of the cases corresponded to patients who had received previous treatment. Patients belonging to the subsidized regime (paid by the government) accounted for 64.66% of the cases. Coinfection with HIV was reported in 12.73% (table 2).

**Table 2:** Demographic and clinical characteristics of MDR-TB cases and controls, Colombia, 2013-2018

Variable		Cases		Controls		P
		n	%	n	%	
Sex						
	Male	382	63.99	892	61.60	0.3138
	Female	215	36.01	556	38.40	
Age group (years)						
	1 to 14	9	1.51	28	1.93	0.05441
	15 to 24	94	15.75	252	17.40	
	25 to 34	124	20.77	283	19.54	
	35 to 44	123	20.60	234	16.16	
	45 to 54	100	16.75	304	20.99	
	55 to 64	94	15.75	197	13.60	
	≥ 65	53	8.88	150	10.36	
Ethnicity						
	Indigenous	38	6.37	86	5.94	0.005062
	Black, Afro	76	12.73	110	7.60	
	Raizal	1	0.17	4	0.28	
	Other	479	80.23	1241	85.70	
	Palenquero	1	0.17	0	0.00	
	ROM	2	0.34	7	0.48	
Health insurance regime						
	Contributory	211	35.34	498	34.39	0.6812
	Subsidized	386	64.66	950	65.61	
Case according to treatment history						
	New	240	40.20	1448	100.00	<0.0001
	Previously treated	357	59.80	0	0.00	
Weight level according to BMI					0.00	
	Low weight	205	34.34	413	28.52	0.03329
	Normal	296	49.58	802	55.39	
	Overweight	77	12.90	166	11.46	
	Obese	19	3.18	57	3.94	
Presence of risk factors						
	Yes	316	52.93	531	36.67	< 0.0001
	No	177	29.65	917	63.33	
	No data	104	17.42	0	0.00	
Presence of comorbidities						
	Yes	278	46.57	197	13.60	< 0.0001
	No	319	53.43	1251	86.40	
HIV status						
	Positive	76	12.73	196	13.54	< 0.0001
	Negative	420	70.35	683	47.17	
	No data	101	16.92	569	39.30	

### Risk factors

The univariate analysis showed that the presence of comorbidities contributed to MDR-TB (OR=5.534; CI: 4.413-6.938 p<0.005). In total, 278 patients reported having some type of comorbidity, malnutrition being the most frequent at 116 cases (19.43%), followed by diabetes at 77 cases (12.90%) and HIV at 73 cases (12.23%). Less frequent comorbidities were silicosis, kidney disease, liver disease, cancer, and arthritis.

Another factor that contributed to MDR-TB was having been exposed to at least one of the following conditions: being drug dependent, taking an immunosuppressant, living in areas with a high burden of drug-resistant tuberculosis (OR=3.083; 95% CI: 2.478-3.838, p<0.005). Three hundred sixteen patients reported at least one of the conditions, the most frequent being living in high-burden areas, at 196 cases (32.83%), drug dependence at 84 cases (14.07%) and taking immunosuppressive medication at 78 cases (13.07%).

Belonging to the black, or Afro ethnicity, being between 35 and 44 years old, being underweight, and having had a previous hospitalization for tuberculosis were variables that also showed an association with MDRTB (table 3).

**Table 3:** Univariate logistic regression analysis of the factors related to multidrug-resistant tuberculosis, Colombia, 2013-2018

Variable		OR	95% CI		P
Male sex		1.1	0.904	1.357	0.3118
Patient with previous hospitalization		1.257	1.017	1.552	0.029
Contributory health insurance regime		1.042	0.849	1.279	0.6812
New patient		0.004	0.002	0.0085	< 0.005
Low BMI		1.31	1.062	1.614	0.0092
Normal BMI		0.792	0.651	0.963	0.0167
Presence of some risk factor		3.083	2.478	3.838	< 0.005
Presence of comorbidities		5.534	4.413	6.938	< 0.005
Prison population		0.509	0.268	0.907	0.0174
Age group (years)					
	1 to 14	0.776	0.320	1.704	0.5109
	15 to 24	0.886	0.677	1.155	0.3633
	25 to 34	1.079	0.844	1.374	0.5277
	35 to 44	1.346	1.046	1.726	0.0161
	45 to 54	0.757	0.583	0.976	0.0284
	55 to 64	1.186	0.898	1.559	0.2078
	≥ 65				
Ethnicity		0.843	0.594	1.181	0.3084
	Indigenous	1.076	0.705	1.617	0.7137
	Black, mulatto, Afro	1.774	1.283	2.442	0.0002
	Other population groups	0.677	0.524	0.877	0.0021

The multivariate analysis confirmed that the presence of at least one of the comorbidities or risk conditions, as well as being of black, Afro, race, was associated with MDR-TB (Nagelkerke’s R2= 0.3013; x^2^ = 662.81; p<0.005) (table 4).

**Table 4:** Multivariate logistic regression model to explain the risk factors for MDR/RR-TB, Colombia, 2013-2018

Variable		OR	95% CI	*p*
Explanatory variables (R^2^ = 0.3013)				
	Presence of at least one comorbidity	907.96	126.4-6521.88	< 0.005
	Presence of at least one risk factor	2.331	1.800-3.020	< 0.005
	Black, Afro	1.924	1.313-2.818	0.010
	Age 35 to 44 years	1.213	0.873-1.684	0.248
	Low BMI	0.746	0.553-1.006	0.055
	Prior hospitalization fo tuberculosis	0.965	0.724-1.288	0.813

OR: Odds ratio; 95% CI: 95% confidence interval; *p*: Wald test; significance level p<0.05

## Discussion

The care of cases of MDR-TB in Colombia is reported by health service provider institutions guided by the Ministry of Health and Social Protection for the programmatic management of drug resistance ^11^^,^^12^. Although these guidelines have existed since 2013, MDR-TB cases have shown an increasing trend, especially during 2017 and 2018 because territorial entities have strengthened their diagnostic capacity and identified more cases or because the actions carried out for control have not been sufficient and it has not been possible to cut the chain of transmission. Higher proportion of new MDRTB cases reflects the actions of tuberculosis control, the conditions, and the effectiveness of the treatment. The actions of the tuberculosis program in each territorial entity should aim to ensure treatment for cases of sensitive tuberculosis and strengthen the management of drugs as well as the programmatic management of multidrug-resistant tuberculosis following the guidelines established ^11^^-^^16^.

The highest rates were found in males between 25 and 54. This finding is consistent with the global behavior of tuberculosis that mainly affects men and young people, as described in countries defined by the WHO as having a high burden for MDR-TB all over the world ^3^. Studies in other contexts have also reported age less than 40 years as a risk factor for MDR-TB ^14^. In Bangladesh and China, the highest number of MDR-TB cases is in the population aged 18 to 45 years and 25 to 44 years, respectively ^15^^,^^17^. The impact of MDR-TB on young people has strong socioeconomic implications due to the loss of productivity and the financial burden it generates for patients and their families ^16^. Patients with MDR-TB spend between 67 and 100% of their annual income to treat their disease ^3^

In our study, a high proportion of the cases corresponded to patients who had received previous treatment. Many studies have found that the most important risk factor for MDR-TB is a history of previous treatment. A study in Sudan showed that failure of previous treatment and living in rural areas were predictors of MDR-TB. These risk factors were related to problems of access, adherence to treatment, and lifestyle ^18^^,^^19^ . In accordance with these results, SIVIGILA reported this risk factor in 40% and 36.6% of tuberculosis cases for the year 2019 and 2020, respectively ^4^, which should alert the health system about the early detection of MDR-TB cases.

In this study, more than half of MDR-TB cases occurred in people affiliated with the subsidized health regime, a model where services and care are provided by state resources when the affiliate does not have a formal job that allows them to contribute to the health system ^20^. According to data from the National Department of Statistics (DANE) in Colombia, only 24% of those affiliated with the subsidized health regime had some occupation ^21^. This reflects the social and economic vulnerability of people with MDR-TB who should be treated by the national government and local governments following the guidelines for the care of the social determinants of health indicated in the public policy of comprehensive health care in Colombia ^22^. On the other hand, the pandemic has affected access to health services for tuberculosis, especially in the poorest population, which indicates the strengthening of universal health coverage as a key factor for tuberculosis and all diseases ^23^.

Other risk factors vary according to the setting but can include hospitalization, incarceration, comorbidities, and HIV infection. Like other authors, we found in the univariate analysis that previous hospitalization of patients with tuberculosis is associated with MDR-TB ^24^. It is likely that a hospitalized patient faces greater complications of their disease than outpatients, which could lead to the development of drug resistance. Even so, when adjusting the multivariate analysis, no association was found between previous hospitalization and MDR-TB.

The association between low BMI and MDR-TB has been described previously. This link is explained by the presence of a weak immune system, which means that the disease cannot be controlled or is reactivated. A negative linear relationship between BMI and tuberculosis has been reported ^25^. In our study, although low BMI was at first associated with MDR-TB, after adjustment for confounders, this association disappeared.

The presence of comorbidities contributes to the presence of MDR-TB. The meta-analysis carried out by Tegegne *et al.*^26^ found that in 24 observational studies from 15 different countries, diabetes had a significant association with MDR-TB (OR=1.97; 95% CI: 1.58-2.45; I^2^= 38.2%), which association was maintained regardless of the level of income of the country, the type of diabetes mellitus, or the design of the study ^26^. Other studies have also reported diabetes as the main risk factor for MDR-TB, which is consistent with our findings and was included among the comorbidities ^18^. Gómez *et al.* found in Mexico that among the comorbidities associated with MDR-TB, malnutrition, HIV, and drug abuse showed no association nor any differences from the group of patients with non-MDR-TB, whereas previously treated cases showed a strong association ^27^. It is important to mention that the rate of diabetes in Mexico and Colombia is high, with the disease occurring in 10.4 and 8.0% of the population, respectively ^28^^,^^29^.

Many publications have found that HIV is not a risk factor for the development of resistance to tuberculosis ^14^^,^^30^^,^^31^, though contradictory data have been described. Sultana *et al*. ^32^ found that the combined odds of MDR-TB were 1.42 times higher in people living with HIV than in HIVnegative patients (OR=1.42; CI=1.17-1.71; I^2^=75.8%), indicating that HIV infection increases the risk of MDR-TB. Similarly, Faustine *et al*. found that in European countries, patients with MDR-TB were more likely to be HIVpositive (OR=3.52; 95% CI: 2.48 to 5.01) ^33^. Tuberculosis is an important cause of death in patients with HIV. In this study, the proportion of patients with coinfection was 2.5%, which was lower.

In our study, except for the presence of comorbidities, once confounders were adjusted for MDR-TB was associated with ethnicity (black race, and Afro-descent) and one or more other risk factors. Belonging to the black, or Afro ethnic group was a risk factor for MDR-TB. In Colombia, 9.34% of the total national population belongs to this ethnic group ^34^. This may be related to the vulnerability factors present in this population. The multidimensional poverty index for this population group that includes educational, work, housing, and access to health services is 30.6%, 11 points above the national index ^35^. This may explain the greater involvement of MDR-TB in this population.

Some limitations are not having collected information on smoking and alcohol consumption, because these variables are not routinely collected within the surveillance system. It was not possible to analyze all the variables that are collected by the surveillance system, mainly due to multiple missing data, especially in the controls. It is necessary to strengthen the collection of information in the primary data generating units.

The presence of comorbidities, the existence of at least one risk factor, such as the ethnic groups of black and Afro-descent, drug dependence, taking immunosuppressive medications, living in an area of high burden of drug-resistant tuberculosis are the main risk factors associated with MDR/ RR-TB. Despite limitations in the study design and information retrieval, these findings will be relevant at the time of diagnosis of tuberculosis. The clinician should determine if the patient has one of these risk factors to decide whether to perform the laboratory analysis of resistance to rifampicin and isoniazid before initiating anti-tuberculosis treatment.

Additionally, tuberculosis programs at the local level should strengthen the routine and detailed monitoring of patients with tuberculosis to find and treat possible cases of MDR-TB in a timely manner, mainly in hospitalized patients of low weight who suffer some comorbidities, are drug addicts, take immunosuppressive medications, live in areas with a high burden of drug-resistant tuberculosis, as well as if they belong to the black, or Afrodescendant ethnic group in Colombia.
